# How Prepared Are the Health Care Professionals for Disaster Medicine Management? An Insight from Pakistan

**DOI:** 10.3390/ijerph19010200

**Published:** 2021-12-25

**Authors:** Ali Hassan Gillani, Shi Li, Jamshaid Akbar, Sumaira Omer, Bakhtawar Fatima, Mohamed Izham Mohamed Ibrahim, Yu Fang

**Affiliations:** 1 Department of Pharmacy Administration and Clinical Pharmacy, School of Pharmacy, Xi’an Jiaotong University, Xi’an 710061, China; hassangillaniali@yahoo.com (A.H.G.); 15802421326@163.com (S.L.); sammaraomer@gmail.com (S.O.); 2 Center for Drug Safety and Policy Research, Xian Jiaotong University, Xi’an 710061, China; 3 Shaanxi Centre for Health Reform and Development Research, Xian Jiaotong University, Xi’an 710061, China; 4 Department of Pharmaceutical Sciences, Superior University Lahore, Lahore 54000, Pakistan; dr.jamshaidsheikh@gmail.com; 5 Department of Obstetrics and Gynecology, King Edward Medical University, Lahore 54000, Pakistan; Drbakhtawar59@gmail.com; 6 Department of Clinical and Pharmacy Practice, College of Pharmacy, QU Health, Qatar University, Doha 2713, Qatar; mohamedizham@qu.edu.qa

**Keywords:** disaster, disaster preparedness, healthcare personnel, low- and middle-income countries, healthcare settings

## Abstract

Countries need healthcare professionals who are competent first responders with a positive attitude and prepared to deal with catastrophes. The study evaluated the knowledge, attitude, and readiness of the practice of healthcare professionals towards disaster management. A survey was carried out among hospital healthcare professionals using a self-administered validated questionnaire. The questionnaire comprised knowledge, attitude, and readiness to practice items. Descriptive and inferential statistics (Mann–Whitney, Kruskal–Wallis, correlation and regression tests) at alpha = 0.05 were used in the analysis. The mean (SD) score of knowledge was 12.25 (4.27) (range: 3.00 to 20.00), attitude (39.32 ± 9.55; range: 18.00 to 61.00), readiness to practice (32.41 ± 6.69; range: 21.00 to 61.00), and KArP (83.99 ± 12.21; range: 60.00 to 124.00). The average knowledge score was moderate, low attitude score, moderate readiness to practice score, and an average score of overall KArP. Attitude is a significant predictor of readiness to practice (*p* = 0.000). The levels of knowledge, attitude, and readiness of the practice of healthcare professionals were not satisfactory. The educators and health policymakers should build a robust curriculum in disaster medicine management and preparedness to prepare for the future of competent healthcare professionals for the nation.

## 1. Introduction

In this contemporary era, catastrophic events are becoming ubiquitous, causing a more detrimental and pronounced impact on societies’ health, quality of health services, the structure of health care systems, and countries’ economies [1]. The operational readiness of health care facilities plays a pivotal role in public health safety [1]. In the past few years, the severity and rate of human-made and natural disasters have escalated globally, putting humankind’s survival in jeopardy [2,3,4]. One of the recent examples is the COVID-19 pandemic, which has caused many deaths worldwide. One must ask whether the health care systems are sufficiently developed to cope with sudden hazardous events.

Pakistan is prone to natural disasters due to its physiographical features and climatic extremes [5]. More significantly, some hazards such as landslides and floods happen annually, whereas other hazards such as earthquakes and tsunamis occasionally appear [5]. However, all these disasters are highly destructive and responsible for devastating results [5]. In 2015, the Hindukush earthquake caused 272 deaths and 2123 injuries. Besides this, Pakistan has been hit by several floods. Among these floods, the most serious was the flood in the year 2010, which caused approximately 1200–2200 deaths [6]. The National Disaster Management Authority (NDMA) was organized in 2007 to protect the state against all these calamities [5].

Nevertheless, the performance of NDMA is not satisfactory as no appropriate contingency planning has been executed to control these unexpected events. One recent example is COVID-19 rapidly escalating in Pakistan due to a lack of expertise, knowledge, and readiness to practice disasters among medical staff [7]. Finally, in June 2020, the number of infected cases surpassed China, making it a health care system failure for Pakistan [8].

Health care professionals (HCPs) play a vital part in managing disasters, including physicians, pharmacists, and nurses. These personnel makes significant decisions in emergency conditions because they act as part of the health care force. More importantly, countries’ proper response in case of disaster is typically based on the qualification, collaboration, and assessment skills of these HCPs. Therefore, the hospital’s firmly established preparedness program guarantees effective management for sudden emergencies [1]. A hospital’s proficiency to tackle a disaster could be estimated by the programs and systems developed before the emergency circumstances [1]. These programs should be arranged to cater to all the essential medical needs of the victims, simultaneously alleviating the negative consequences of specific events on health services [1]. Evaluation of readiness for hazardous events and activeness in response to handling this event could be a facile means to distinguish the possible gaps and limitations of the hospital to manage hazardous events in real-time [9,10,11].

Despite the fact that WHO has published the hospital safety index, a checklist for the assessment of hospital preparedness [12], which is standardized and recognized globally is required [13,14,15]. However, so far, there are no internationally regulated legal standards for assessing hospital preparedness [16]. Pakistani Doctors were the part of many teams for rescue missions around the world so as to playing part in the global disaster management these healthcare professionals should be well organized and trained in terms of disaster medicine [17,18]. Thus, this research aims to identify the level of disaster medicine preparedness of Pakistani HCPs, mainly their knowledge (K), attitude (A), and readiness to participate (rP) in catastrophic events. The research also proposes recommendations that would indeed foster the skills of HCPs to manage future unforeseeable events efficiently.

## 2. Materials and Methods

### 2.1. Study Design

A cross-sectional, survey-based study was carried out among the HCPs of hospitals, primary healthcare centers, community pharmacies, and clinics in Lahore and Bahawalpur cities, located in the Punjab province of Pakistan. The study period was two months, from July to August 2020.

### 2.2. Ethical Considerations

Study approval was given by the institutional review committee of Xi’an Jiaotong University (Ref# DIS 2-2020) and further approved by the Superior University Lahore. Besides this, verbal consent was also obtained after explaining the important aspects of the research (objective, importance, benefits) to all study participants. The data was kept confidential and used only for research purposes. Participants were well informed that they have the autonomy to leave the study and their names were also not determined on the questionnaires. More importantly, the questionnaire was anonymous.

### 2.3. Study Participants and Sampling

The study population was enlisted from different services/departments of the hospitals: the Jinnah Hospital Lahore, Ganga Ram Hospital Lahore, Masood Hospital Lahore, Chaudhry Muhammad Akram Teaching and Research Hospital Lahore, Bahawal Victoria Hospital Bahawalpur, and Civil Hospital Bahawalpur, in addition to primary healthcare centers, private clinics, and pharmacies. All paper-based surveys were handed out anonymously during departmental staff meetings and requested to fill at the spot. The sample size was estimated using Raosoft based on a 5% margin of error and considering a 95% confidence level and a response rate of 80%. The estimated sample size required was 357. Participants were selected conveniently and included in the research with no limitations on gender, age, or experience. Those who were not willing to participate were excluded from the study.

### 2.4. Tool Development and Quality Measures

The questionnaire was developed after an extensive literature review. A pre-validated questionnaire was utilized [19,20]. Participants were assessed on three primary parameters: knowledge, attitude, and readiness to practice (KArP). Section one is equipped with the demographic details, e.g., age, gender, and occupation. Section two of the questionnaire was designed to evaluate participant’s knowledge of disaster management. This part consisted of 22 closed-ended binary questions with a range of 0–22 points. Cut-off values were set for categorization of the scores. The points below seven were considered low (25th quartile), scores between 7 to 12 points were taken as moderate (25–75th quartiles), and those scores which surpassed 12 points were termed as high (>75th quartile). The attitude section comprised 16 Likert scale questions (strongly agree, agree, neither agree nor disagree, disagree, and strongly disagree). The maximum score for this part was 80 points, and the minimum was 16 points. Those scores that fall behind 42 were taken as low, points that were in the range of 42–56 were tagged as moderate (25–75th quartiles), and those exceeding 56 were scored as high. The final section was based on readiness to practice having 11 Likert scale questions (strongly agree = 1, agree = 2, neither agree nor disagree = 3, disagree = 4 and strongly disagree = 5 and Not applicable = 6) that could get a sum of 55 points. Score less than 31 points, 31–38 points, and more than 38 points were designated as low (25th quartile), moderate (between 25th to 75th quartile), and high (75th quartile), respectively. A pilot study was also conducted with 30 HCPs before the data collection. Minor adjustments were made in response to the participant feedback to develop the most appropriate survey tool. Cronbach’s alpha measures indicated that the knowledge domain = 0.781, attitude domain = 0.854, and the readiness to practice domain = 0.646.

### 2.5. Data Collection Procedures

A self-administered survey was conducted to collect data from the HCPs of the selected institutions. The convenience sampling method is used to approach the respondents. A representative from each institutions was selected to approach the respondents to increase participation.

### 2.6. Data Analysis

Data analysis was done using SPSS version 26 (IBM Corp. Released 2018. IBM SPSS Statistics for Windows, Version 26.0. Armonk, NY, USA). The Kolmogorov–Smirnov test was applied to check the normality of the data. Descriptive statistics, including frequency (%) for categorical variables, were used, whereas mean or median for continuous variables. Since the normal distribution of data was not found during analysis, nonparametric tests (i.e., Kruskal–Wallis and Mann–Whitney) were employed. Pearson’s correlation test was also done to find the correlation between the three parameters (K, A, and rP). Linear regression was carried to predict the relation between the readiness to practice and knowledge, and attitude. A priori level of significance was 0.05.

## 3. Results

The average age (mean ± sd) of the respondents was 29.9 ± 5.8. Further findings below (Table 1) indicate that most of the respondents were female (n = 228, 60.5%), nurse (n = 146, 38.7), located in Lahore city (63.4%), worked in a hospital setting (n = 319, 84.6%) and median (IQR) working experience of 4.0 (2.0–6.0) years. Further analysis indicated that the mean (sd) score of knowledge was 12.25 (4.27) (range: 3.00 to 20.00), attitude (39.32 ± 9.55; range: 18.00 to 61.00), readiness to practice (32.41 ± 6.69; range: 21.00 to 61.00), and KArP (83.99 ± 12.21; range: 60.00 to 124.00).

Table 2 below illustrates the knowledge viewpoint of the respondents. A high percentage of respondents agreed that Disasters come in many shapes and sizes (88.3%); Disaster medicine is genuinely a systems-oriented specialty and involved multiple responding agencies (83.0%). Realistic on-scene training is vital to an efficient and effective disaster medicine plan (78.5%). On the other hand, 78.2% of the respondents disagreed with the statement “I do not think Pakistan is at risk of disasters (natural or human-made)”. More perspectives can be found below.

Table 3 below demonstrates respondents’ attitudes regarding preparedness for medicine-related disasters. Three top viewpoints that indicated agreement were: I would be interested in educational classes on medicine-related disaster preparedness that relates specifically to the country situation (88.1%); I would be willing to be a future member of a healthcare response team in case of a medicine disaster (77.2%); and as a healthcare professional, I would feel confident in my abilities as a future healthcare provider and first responder in a disaster medicine situation (70.3%).

The respondents’ readiness to practice was showed in Table 4 below. Among the three highest agreement standpoints were: I am willing to attend the emergency medicine education incorporated in the continuous professional education program (83.6%); I need to be more trained on providing patient-centered care under the situation of disaster medicine (75.6%), and it requires effort and time to be prepared (73.4%).

Differences in total knowledge, attitude, and readiness to practice scores in terms of the respondents’ demographic characteristics were presented in Table 5. Age is significantly associated with readiness to practice (*p* = 0.016). The profession of the respondents was related to the knowledge (*p* = 0.000), attitude (*p* = 0.000), and the overall KArP (*p* = 0.003), except the readiness to practice (*p* = 0.068). Further findings showed that the respondents’ location, present workplace, and working experience were related to knowledge scores and attitude scores.

Table 6 demonstrates the cause-effect relationship between the different factors and readiness to practice of the respondents. The significant predictor was the total score for attitude items (*p* = 0.000).

## 4. Discussion

This study aimed at exploring knowledge, attitude, and readiness to the practice of healthcare professionals towards disaster management in Pakistan. The average knowledge score of the respondents was moderate, low attitude score, moderate readiness to practice score and an average score of overall KArP. Attitude is a significant predictor of readiness to practice.

Due to threats and the complex nature of the disaster, hospitals and other healthcare delivery organizations must be prepared to care for those in need of medical services and protect individuals from being exposed to any further risk [21]. Our study demonstrated that 78.2% of the professionals think that our country is prone to disasters which is in line with the previous study from Yemen where 85.5% of the respondents responded the same [22]. Additionally, in our study, 45.1% said that they know who to contact in case of disaster this was higher than the results observed from the study in Jordan (35.5%) [23]. WHO emphasized that hospitals and other healthcare facilities should play a critical role in national and local emergency responses [24]. According to Chaffee and Oster (2006), the impact of disaster is shocking. The hospital and other healthcare institutions and the personnel must be prepared for the challenges [25]. They must have the knowledge, positive attitude and readiness to practice towards disaster. The community expects the healthcare staff to be available to provide care for them during the catastrophic. During a disaster, staff may become either responder or victim, or both. Thus, their ability and willingness to work are crucial [26]. However, in our results, 57.0% showed that finding relevant information about disaster medicine preparedness related to this country’s needs is an obstacle to their level of preparedness this is high compared with the results found in Jordan (28.0%) [23].

Our study findings indicated that the knowledge was moderate. In a study in Saudi Arabia, Nofal et al. (2018) evaluated hospital staff’s knowledge, attitudes, and practices about disaster and preparedness. They reported that the knowledge level of physicians and nurses was satisfactory [27]. A study in Iran showed that the nurses’ knowledge was moderate [28]. The authors also found a significant relationship between knowledge score and respondent’s age and job experience. One study in Malaysia was carried out to evaluate the knowledge of medical personnel [29]. The majority of them have adequate knowledge of disaster management. Naser and Salem (2018) studied the healthcare professional who is considered first responder knowledge and attitude towards disaster preparedness [30]. Their overall knowledge was insufficient. According to them, this poor knowledge was due to a lack of teaching programs. Generally, these studies illustrated inadequate levels of knowledge among healthcare staff.

In terms of attitude, our study showed that the attitude of the respondents was low and 88.1%were agreed/strongly agreed in showing the interest in educational classes on disaster medicine preparedness that relates specifically to their country situation these results were in consistent with the previous Yemeni study, where 82.5% showed the interest in the educational activities [22]. Nofal et al. (2018) discovered that the attitude of the hospital staff in Saudi Arabia was neutral [27]. Far et al. (2020) indicated that the level of attitude of the nurses in Iran was just moderate [28]. Ahayalimudin and Osman (2016), in their study findings in Malaysia, showed that the physicians, nurses, and assistant medical officers’ attitudes towards disaster management were optimistic [29]. A study in Yemen illustrated that healthcare professionals’ attitudes were surprisingly positive [22]. Our study also reported that 43.2% had agreement with family members on how to execute our personal/family emergency and disaster medicine plans; this is also found in a Jordanian study, where only 30.2% stated the same [23]. All these findings showed that the level of attitude of the healthcare personnel was between low and satisfactory.

The readiness to practice level of our respondents was moderate. Nofal et al. (2018) discovered that the practice of the hospital staff was neutral [27]. Far et al. (2020) studied the performance of the nurses related to disaster management and mentioned that it was moderate, and gender, age, marital status, and job experience were significantly related to the performance aspect [28]. In another study, the researchers found out that the practices among medical staff were adequate [29]. The staff’s working experience and training in disaster management programs were associated with higher positive scores. Overall, these studies illustrated a moderate level of readiness to practice among the healthcare staff. Our study determined that the readiness to practice can be strongly influenced by the staff attitude (Beta 0.309, 95%CI 0.230–0.388 *p* < 0.001); this is similar to the previous results of students in Pakistan and Qatar [19,20]. Thus, we strongly believe the importance of improving the attitude and enhancing competency may strengthen the level of readiness to practice during disaster.

## 5. Strength and Limitations

This study has a few limitations. One of the limitations is that the results are related to Pakistani culture, environmental, and educational context, which may not be in consistency with other countries. Therefore, we cannot generalize the findings to other LMICs. This study targeted only few geographical areas of Pakistan, but Pakistan has a wider area and more critical geographical areas such as Kashmir, KPK. The study should be carried out in other regions. However, because the study relied on a survey, self-reported data and self-perception might have caused biases. In contrast, this study contributes another important perspective from healthcare professional in LMICs.

## 6. Recommendations

Relevant stakeholders related to disaster management in the country need to ensure healthcare facilities and their staff are staffed with skilled, competent personnel who can provide quality care to individuals. Thus, university colleges and continuous education programs for HCPs should consider incorporating theoretical and practical components on disaster and preparedness in the curriculum [27,29]. The quality of these programs and curriculum according to the need assessment are also important [28]. Moreover, healthcare institutions need to ensure the effectiveness of human resource management [30]. The management must ensure enough personnel capacity for continuity of operations during disaster events. The training and exercises must be regularly carried out. These sessions may include modules and operational simulations related to the aspect of medicines and other pharmaceutical items. These items must be available and accessible during emergencies [28]. It should be part of the disaster response plan.

## 7. Conclusions

In summary, this study illustrated that the healthcare professionals in our study have a moderate level of knowledge, a low attitude level, a moderate readiness to practice level, and an average level of overall KArP. Attitude is a significant predictor of readiness to practice. We strongly believe that educators and health policymakers should build a robust curriculum in disaster medicine management and preparedness for the future of competent healthcare professionals of the nation.

## Figures and Tables

**Table 1:** Demographic profiles of the healthcare professionals.

Characteristics	Subitem	Mean (SD)	Median (IQR)	Frequency (*n*)	Percentage (%)
Age		29.9 (5.8)	29.0 (26.0–32.0)		
Gender	Female			228	60.5
	Male			149	39.5
Profession	Pharmacist			40	10.6
	Physician			134	35.5
	Dentist			14	3.7
	Nurse			146	38.7
	Others			43	11.4
Locality	Lahore			239	63.4
	Bahawalpur			138	36.6
Present workplace	Hospital			319	84.6
	Primary healthcare			8	2.1
	Community pharmacy			19	5.0
	GP clinic			16	4.2
	Others			15	4.0
Working experience		5.0 (5.3)	4.0 (2.0–6.0)		

**Table 2:** Knowledge assessment of the respondents regarding disaster medicine preparedness.

Statement	Yes	No
1. I have previous exposure to this topic (*Disaster Medicine Preparedness*).	104	273
2. I have previous experience in dealing with disasters.	127	250
3. I do not think Pakistan is at risk of disasters (natural or human-made).	82	295
4. Disasters come in many shapes and sizes.	333	44
5. Disaster medicine is the sole responsibility of a pharmacy organization.	259	118
6. I read journal articles related to medicine disaster preparedness.	114	263
7. I am not aware of programs about disaster medicine preparedness and management offered, for example, at either my workplace or community.	108	269
8. I find that the research literature on disaster medicine preparedness and management is not easily accessible.	111	266
9. I find that the research literature on disaster medicine preparedness is understandable.	243	134
10. Finding relevant information about disaster medicine preparedness related to this country’s needs is an obstacle to my level of preparedness.	215	162
11. I know where to find relevant research or information related to disaster medicine preparedness and management to fill in gaps in my knowledge.	216	161
12. I know referral contacts in case of a disaster medicine situation (e.g., health department).	170	207
13. In case of a disaster medicine situation, I think there is sufficient support from local officials on the governance level.	150	227
14. I am aware of the potential risks emergencies in Pakistan are (e.g., natural disaster, embargo, terror, war, etc.).	232	145
15. I know how such emergencies or disasters can affect the medication supply system (selection, quantification, procurement, storage, distribution).	274	103
16. I know the limits of my knowledge, skills, and readiness as healthcare personnel to act in disaster medicine situations, and I know when I exceed them.	287	90
17. In the case of the war, I know how to overcome the access to medicines problem to benefit my society.	243	134
18. I am familiar with the local emergency response system for medical disasters.	214	163
19. I am familiar with the accepted process of ‘examining problems to decide which ones are the most serious and must be dealt with first (triage principles)’ used in disaster medicine situations.	219	158
20. I am familiar with the organizational logistics and roles among local and national agencies in disaster medicine response (i.e., taking decisions and measures).	186	191
21. Realistic on-scene training is vital to an efficient and effective disaster medicine plan.	296	81
22. Disaster medicine is genuinely a systems-oriented specialty and involved multiple responding agencies.	313	64

**Table 3:** Attitude assessment of the respondents regarding disaster medicine preparedness.

	Strongly Agree	Agree	Neither Agree Nor Disagree	Disagree	Strongly Disagree
1. I consider myself prepared for the management of disaster medicine.	88 (23.3)	141 (37.4)	73 (19.4)	73 (10.4)	2 (0.5)
2. I would feel confident in my abilities as healthcare personnel in disaster medicine situation.	36 (9.5)	69 (18.3)	89 (23.6)	139 (36.9)	44 (11.7)
3. I would be interested in educational classes on disaster medicine preparedness that relates specifically to the country situation	156 (41.4)	176 (46.7)	31 (8.2)	14 (3.7)	0 (0.0)
4. I would be considered a key leadership figure in my community in a disaster medicine situation.	85 (22.5)	169 (44.8)	51 (13.5)	56 (14.9)	16 (4.2)
5. I have personal/family emergency plans in place for disaster medicine situations.	69 (18.3)	87 (23.1)	78 (20.7)	114 (30.2)	29 (7.7)
6. I have an agreement with loved ones and family members on how to execute our personal/family emergency and disaster medicine plans.	55 (14.6)	108 (28.6)	72 (19.1)	111 (29.4)	31 (8.2)
7. I cannot describe my role in the response phase of disaster medicine in the context of my workplace, the general public, media, and personal contacts.	11 (2.9)	92 (24.4)	119 (31.6)	123 (32.6)	32 (8.5)
8. I would feel confident as a future manager or coordinator of a shelter/healthcare/medication supply facility.	72 (19.1)	179 (47.5)	92 (24.4)	30 (8.0)	4 (1.1)
9. I would be willing to be a future member of a healthcare response team in a medicines disaster.	101 (26.8)	190 (50.4)	44 (11.7)	38 (10.1)	4 (1.1)
10. I feel reasonably confident I can care for patients independently without supervision in a medicines disaster situation.	76 (20.2)	175 (46.4)	57 (15.1)	67 (17.8)	2 (0.5)
11. I would feel confident implementing emergency and disaster medicine plans and procedures.	87 (23.1)	147 (39.0)	71 (18.8)	70 (18.6)	2 (0.5)
12. I would feel confident in providing medicine-related education in case of disaster or emergency.	93 (24.7)	161 (42.7)	76 (20.2)	31 (8.2)	16 (4.2)
13. As a health personnel, I consider myself prepared for the management of medical disasters.	94 (24.9)	96 (25.5)	116 (30.8)	69 (18.3)	2 (0.5)
14. As a health personnel, I would feel confident in my future healthcare provider and first responder in a disaster situation.	68 (18.0)	197 (52.3)	49 (13.0)	52 (13.8)	11 (2.9)
15. There is enough awareness on “ways to stand wars and other human and natural emergencies” among healthcare personnel in the workplace	57 (15.1)	128 (34.0)	69 (18.3)	87 (23.1)	36 (9.5)
16. I do not need more workshops and simulated training to be ready for dealing with disaster medicine.	25 (6.6)	34 (9.0)	72 (19.1)	158 (41.9)	88 (23.3)

**Table 4:** Readiness to practice assessment of the respondents regarding disaster medicine preparedness.

	Strongly Agree	Agree	Neither Agree Nor Disagree	Disagree	Strongly Disagree	Not Applicable
1. My role in disaster medicine situations is clear.	54 (14.3)	162 (43.0)	51 (13.5)	78 (20.7)	14 (3.7)	18 (4.8)
2. I am not ready to handle whatever potential risks emergencies exist in the community.	5 (1.3)	72 (19.1)	54 (14.3)	172 (45.6)	30 (8.0)	44 (11.7)
3. I am willing to attend the emergency medicine education incorporated in the continuous professional education program.	140 (37.1)	175 (46.5)	27 (7.2)	12 (3.2)	9 (2.4)	14 (3.7)
4. I attended workshops/seminars about disaster medicine, and it is enough for me to practice in a real situation.	99 (26.3)	89 (23.6)	23 (6.1)	105 (27.9)	16 (4.2)	45 (11.9)
5. My college courses enable me to be ready to practice in the settings of disaster (natural: e.g., earthquakes and floods; or human-made: e.g., embargo or wars)	75 (19.9)	130 (34.5)	66 (17.5)	68 (18.0)	20 (5.3)	18 (4.8)
6. Other extracurricular resources (e.g., internet, TV, radio, and newspapers) enable me with a sufficient degree of readiness to practice under disaster.	31 (8.2)	125 (33.2)	121 (32.1)	68 (18.0)	32 (8.5)	0 (0.0)
7. I am ready to practice under disaster, knowing that some essential medications may not be available because of the disaster situation.	21 (5.6)	63 (16.7)	81 (21.5)	143 (37.9)	43 (11.4)	26 (6.9)
8. I need to be more trained on providing patient-centered care under the situation of disaster medicine.	128 (34.0)	157 (41.6)	45 (11.9)	26 (6.9)	5 (1.3)	16 (4.2)
9. The following are barriers that reduce my readiness to practice:						
○ Lack of knowledge about medication disaster. → being unfamiliar with the new medications appearing during disasters. (The previous few questions are dealing with the same issue).	69 (18.3)	122 (32.4)	87 (23.1)	52 (13.8)	40 (10.6)	7 (1.9)
○ Disasters medicines are unlikely to occur in Pakistan.	38 (10.1)	160 (42.4)	89 (23.6)	26 (6.9)	49 (13.0)	15 (4.0)
○ It requires effort and time to be prepared.	94 (24.9)	183 (48.5)	43 (11.4)	42 (11.1)	15 (4.0)	0 (0.0)

**Table 5:** Differences of total knowledge, attitude, and readiness to practice scores in terms of demographic characteristics of the respondents.

Characteristics	Subitem	Knowledge Score	Attitude Score	Readiness to Practice Score	Overall KArP
Age *	Correlation coefficient	0.047	0.016	−0.124	−0.013
		*p*-value = 0.361	*p*-value = 0.759	*p*-value = 0.016	*p*-value = 0.794
Gender **	Female	*p*-value = 0.175	*p*-value = 0.103	*p*-value = 0.784	*p*-value = 0.721
	Male				
		*p*-value = 0.175	*p*-value = 0.103	*p*-value = 0.784	*p*-value = 0.721
Profession ***	Pharmacist Physician Dentist Nurse Others	*p*-value = 0.000	*p*-value = 0.000	*p*-value = 0.068	*p*-value = 0.003
Locality **	Lahore Bahawalpur	*p*-value = 0.001	*p*-value = 0.002	*p*-value = 0.263	*p*-value = 0.329
Present workplace ***	Hospital Primary healthcare Community pharmacy GP clinic Others	*p*-value = 0.000	*p*-value = 0.048	*p*-value = 0.223	*p*-value = 0.123
Working experience *	Correlation coefficient	0.292	−0.175	−0.078	−0.066
		*p*-value = 0.000	*p*-value = 0.001	*p*-value = 0.129	*p*-value = 0.202

Note: * Spearman rho correlation test; ** Mann–Whitney test; *** Kruskal–Wallis test.

**Table 6:** Predictors of readiness to practice.

Model		Unstandardized Coefficients		Standardized Coefficients	T	Sig.	95.0% Confidence Interval for B	
		B	Std. Error	Beta			Lower Bound	Upper Bound
1	(Constant)	23.541	4.379		5.376	0.000	14.930	32.152
	Gender	0.868	0.812	0.064	1.069	0.286	−0.729	2.464
	Locality	0.869	0.689	0.063	1.261	0.208	−0.486	2.224
	Age	−0.153	0.111	−0.132	−1.386	0.167	−0.371	0.064
	Profession	−0.058	0.316	−0.011	−0.183	0.855	−0.680	0.564
	Working experience	0.014	0.123	0.011	0.117	0.907	−0.228	0.257
	Present workplace	0.152	0.962	0.008	0.158	0.874	−1.739	2.044
	Total score for knowledge items	0.025	0.097	0.016	0.262	0.794	−0.166	0.216
	Total score for attitude items	0.309	0.040	0.441	7.664	0.000	0.230	0.388
Dependent Variable: Total score for readiness to practice								

Regression equation: Total score for readiness to practice = 0.441 (Total score for attitude) +23.541.

## Data Availability

Data will be available on request.
